# Understanding human brain function in real-world environments

**DOI:** 10.1371/journal.pbio.3003210

**Published:** 2025-06-16

**Authors:** Feng Zhou, Benjamin Becker

**Affiliations:** 1 Faculty of Psychology, Southwest University, Chongqing, China; 2 Department of Psychology, The University of Hong Kong, Hong KongChina

## Abstract

Functional MRI has been invaluable in understanding brain function, but findings often remain of limited real-world relevance. This Perspective discusses how neuroimaging in more naturalistic environments may reveal crucial insights into human cognition and social interactions in everyday life.

## Teaser

Functional MRI is invaluable in understanding brain function, but findings are often of limited real-world relevance. Neuroimaging in more naturalistic environments could reveal crucial insights into how the brain processes cognition, emotional experiences, and social interactions in daily life.

**Fig 1:**
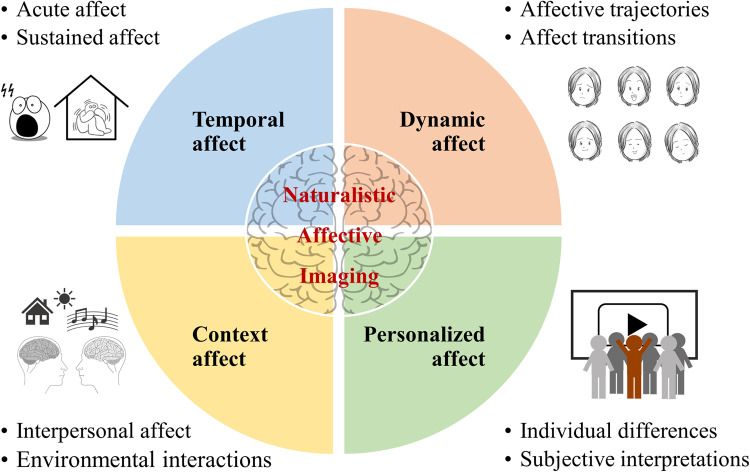
How naturalistic neuroimaging approaches enhance our understanding of affective processes beyond traditional laboratory methods. Temporal affect (blue): contrasts brief, acute emotional experiences with sustained affective states, highlighting how duration influences neural engagement patterns. Dynamic affect (orange): captures continuous affective trajectories and transitions between emotional states. Context affect (yellow): examines how rich interpersonal interactions and person-environment exchanges characterize real-world affective experiences. Personalized affect (green): explores individual variability and subjective interpretations that shape unique affective experiences. The icons in the figure were obtained from pixabay.com under the Pixabay License and are free for use and adaptation for noncommercial purposes.

Functional MRI (fMRI) studies, exploring brain architecture and activity patterns during specific mental processes, have been essential for human neuroscience. Traditionally, task-based fMRI studies employ strictly controlled paradigms with sparsely presented and simplified static stimuli, such as fear-inducing pictures or grayscale faces. These designs have been crucial in validating that neural activity patterns can be reliably attributed to specific cognitive processes, such as recognizing facial emotions while controlling for confounding variables. However, a key assumption is that the findings can be generalized to mental processes in real-world scenarios, enabling conclusions to be drawn about brain function in natural environments, not just in fMRI machines. Recently, ecological validity—that is, the extent to which experimental findings reflect real-world behavior and experience—of neuroimaging experiments has been enhanced by embracing ‘naturalistic neuroimaging’ [1]. This approach has proven particularly powerful for affective neuroscience (Fig 1), the field that investigates how emotional processes are represented and regulated in the brain. The subjective affective experiences emerge through complex interactions between brain, body, and environmental signals, and are modulated through conscious appraisal processes [2]. Beyond its substantial impact in studying affect, the naturalistic paradigm has also yielded important insights in cognitive and social neuroscience research, enabling the establishment of more ecologically valid models of brain processes in dynamic real-world contexts.

An increasing number of neuroimaging studies have utilized naturalistic paradigms (e.g., movie watching) and significant differences between the traditional paradigms and affective processing closer to real-life situations are beginning to emerge. For example, short-term (acute) versus long-term (sustained) affective responses may engage different neural systems [3]. Interestingly, the amygdala—a region frequently identified in studies featuring static threatening images [4] and often described as the brain’s ‘fear center’—is not engaged during dynamic and prolonged fear experience [3]. This region may therefore encode the initial response to potential threats but might not be sufficient to encode sustained and conscious fear [5]. This distinction may be particularly relevant for understanding mental disorders, which are characterized by the persistence of, or alterations in, emotional processing.

Contemporary research has leveraged sophisticated decoding techniques to develop and validate neuromarkers for affective and cognitive processes, aiming to objectively measure mental states based on neuroimaging data. While these neuromarkers, based on distributed brain activity, can accurately identify the current mental state of participants in response to discrete stimuli, recent evidence suggests that these activity-based neurofunctional signatures cannot effectively track dynamic affective or cognitive trajectories. For instance, recent studies have demonstrated that changes in neural activity alone are insufficient to track dynamic variations in mental processes, including affective experience (during capsaicin-induced tonic pain [6] or horror movie-induced fear [5]) or fluctuations in attentional engagement during narrative processing [7]. This limitation is particularly noteworthy given that our real-world experiences unfold continuously and dynamically rather than within discrete events. These studies subsequently highlight that tracking affective and attentional fluctuations requires accounting for dynamic functional connectivity—the context-dependent communication between brain regions—particularly in naturalistic settings. Moreover, transitions between affective states and their blending, which cannot easily be explored with sparsely presented static stimuli, represent an area of interest given that affective experiences in daily life involve continuous shifts between emotional states, and impaired transitions between emotional states (such as difficulty shifting from negative to positive emotions) are characteristic features of many affective disorders [8].

Another substantial difference lies in the environmental complexity of the processes, in addition to the duration and dynamics of affective experiences. Studies on social interactions emphasize these challenges. Traditional studies commonly present static facial stimuli or single-round economic exchange games, whereas real-life social interactions are fundamentally different: they are dynamic, reciprocal, and embedded within rich environmental contexts. Capturing the complexity of real-world social dynamics requires studying natural and unstructured interpersonal interactions as well as human-environment exchanges as they unfold in ecologically valid settings [9].

Individual differences and context-dependent interpretations fundamentally shape how we perceive and respond to real-world experiences; for instance, in how we process and regulate our affective experiences. Despite growing interest in individualized brain-based therapies for emotional disorders [10], the majority of current neuroimaging research employs population-level analyses that emphasize neural patterns across individuals based on simple stimuli or cognitive engagement, grouped according to normative ratings. Naturalistic paradigms offer a compelling alternative, as their rich stimuli content, contextual flexibility, dynamic narrative progressions, and varying degrees of personal relevance create opportunities to better characterize and understand the neurobiological basis of individual variation in cognitive and affective processing [11]. This approach opens additional venues to determine long-standing and evolving questions in the field. It may facilitate better examination of the neural mechanisms underlying mixed affective experiences—a crucial but understudied aspect of human emotion—as naturalistic contexts often elicit multiple distinct emotions simultaneously or in rapid succession, with each individual’s unique experiences shaping their emotional responses. Moreover, it may enable the identification of disorder-related brain alterations in more ecologically valid settings [12], help to determine the therapeutic potential of novel interventions in close to real-life contexts and enable the exploration of dynamic brain processes that shape interactions with new technology.

Naturalistic neuroimaging presents numerous opportunities to study brain function in near-real-life settings, with certain approaches being particularly crucial for advancing our understanding. Movie paradigms employing complex story lines are especially powerful for examining dynamic cognitive and affective processes. As such, these paradigms allow researchers to track continuous trajectories of mental states and transitions between them: a critical advance given that real-world subjective experiences unfold as continuous stream rather than in discrete events. Personal narratives (e.g., naturalistic free recall and cued retrieval of autobiographical events) hold the opportunity to overcome the lack of self-relevance that is often inherent in the traditional paradigm, and can facilitate investigations of memory and emotional experiences, as well as trauma-related mental disorders. Likewise, the inclusion of immersive virtual reality can facilitate the determination of emotional and cognitive dysregulations in these disorders and holds great potential for determining spatial navigation and interactions between cognitive maps and other mental processes. Moreover, advances in portable neuroimaging technologies, like functional near-infrared spectroscopy, wearable scalp electroencephalography, and optically pumped magnetometers [13], together with hyperscanning techniques [14], may provide unprecedented insights into the neural basis of real-world social interactions and interpersonal processes. While the specific aim of the study will influence the choice of naturalistic paradigm, all these approaches will require a careful consideration of the trade-off between ecological validity and experimental control. Each approach presents specific challenges: movie paradigms limit participant interaction but maintain experimental control; personal narratives maximize ecological validity but may lead to large inter-individual variations; virtual reality balances realism with control but may induce motion artifacts and cybersickness (especially in MRI systems); and portable technologies offer mobility at the cost of spatial resolution compared with traditional fMRI. Furthermore, these naturalistic approaches necessitate sophisticated analytical methods to handle the complexity of the data.

Several techniques have been developed to analyze such naturalistic neuroimaging data. For instance, intersubject approaches can be used to detect shared neural activity across individuals (intersubject correlation) as well as individual variation (intersubject representational analysis), and hidden Markov models have been applied to identify changes in discrete states based on dynamic patterns of functional connectivity. However, perhaps the most fundamental challenge is the interpretation of observed neural processes in naturalistic contexts. Although highly controlled experiments have been designed to facilitate clear conclusions (e.g., comparing activity in response to smiling versus neutral faces from the same person), the simultaneous variation of multiple factors and high individual variability complicates clear conclusions in dynamic naturalistic designs. This challenge could be addressed by the execution of bridging studies, which systematically connect controlled and naturalistic conditions to validate interpretations across contexts, and by using machine learning approaches that utilize multiple measurements (neural, physiological, behavioral, and subjective data) to decode mental processes in naturalistic settings. For example, combining machine learning with dynamic connectivity has revealed fundamental neural representations and mechanisms that generalize across individuals during affective and cognitive processing in naturalistic environments [5–7].

Neuroimaging in naturalistic or close-to-real-world environments has the potential to reveal crucial insights that conventional paradigms have missed, enhancing our understanding of neural mechanisms underlying social, affective, and cognitive experiences. Such approaches could be transformative in advancing treatment evaluation of affective disorders under more naturalistic contexts.

## Abbreviation

fMRI: functional MRI
